# Preparation of High-Solid Microfibrillated Cellulose from *Gelidium amansii* and Characterization of Its Physiochemical and Biological Properties

**DOI:** 10.4014/jmb.2210.10009

**Published:** 2022-11-17

**Authors:** Min Jeong Kim, Nur Istianah, Bo Ram So, Hye Jee Kang, Min Jeong Woo, Su Jin Park, Hyun Jeong Kim, Young Hoon Jung, Sung Keun Jung

**Affiliations:** 1 School of Food Science and Biotechnology, Kyungpook National University, Daegu 41566, Republic of Korea; 2 Research Center, Honest Co., Ltd., Daegu 41064, Republic of Korea; 3 Institute of Fermentation Biotechnology, Kyungpook National University, Daegu 41566, Republic of Korea; 4 Research Institute of Tailored Food Technology, Kyungpook National University, Daegu 41566, Republic of Korea; 5 Department of Food Science and Biotechnology, Brawijaya University, Malang 65145, Indonesia

**Keywords:** Microfibrillated cellulose, cosmetic ingredient, inflammation, nanotechnology, *Gelidium amansii*

## Abstract

Microfibrillated cellulose (MFC) is a valuable material with wide industrial applications, particularly for the food and cosmetics industries, owing to its excellent physiochemical properties. Here, we prepared high-solid microfibrillated cellulose (HMFC) from the centrifugation of *Gelidium amansii*-derived MFC right after fibrillation. Dispersion properties, morphology, and structural changes were monitored during processing. HMFC has a five-fold higher solid concentration than MFC without significant changes to dispersion properties. SEM images and FTIR spectra of HMFC revealed a stable surface and structure against centrifugal forces. HMFC exhibited 2,2′-azino-bis (3-ethylbenzothiazoline-6-sulfonic acid) (ABTS) radical scavenging activity, although it could not scavenge 2,2-diphenyl-1-picrylhydrazyl (DPPH). Moreover, HMFC inhibited the generation of LPS-induced excessive nitrite and radial oxygen species in murine macrophage RAW264.7 cells. Additionally, HMFC suppressed LPS-induced Keap-1 expression in the cytosol but did not alter iNOS expression. HMFC also attenuated the UVB-induced phosphorylation of p38, c-Jun N-terminal kinase (JNK) 1/2, and extracellular-signal-regulated kinase (ERK) 1/2, as well as the phosphorylation of c-Jun in the immortalized human skin keratinocyte HaCaT cells. Therefore, the application of centrifugation is suitable for producing high-solid MFC as a candidate material for anti-inflammatory and anti-oxidative marine cosmeceuticals.

## Introduction

Macroalgae (marine algae) are attracting attention as a potential resource for the food and cosmetics industry [1]. Marine algae are generally classified into three major classes: brown (Phaeophyceae), green (Chlorophyceae), and red algae (Rhodophyceae) [1]. Among the red algae, *Gelidium amansii* comprises over 70% carbohydrate and is a critical resource for materials used in food and cosmetics, such as agar, carrageenan, and cellulose. It is also one of the most abundant red algae in intertidal and subtidal zones [2, 3, 4, 5].

General nanocellulose (NC), a kind of cellulose with nanosized particles in the form of fibrils or crystals, can be classified as microfibrillated cellulose (MFC), nanofibrillar cellulose (NFC), cellulose nanocrystal (CNC), and bacterial nanocellulose (BNC) [2, 6]. Cellulose purity varies depending on the raw material and extraction method. Many protocols have been proposed for cellulose extraction from different kinds of biomass [7]. Cellulose is processed by physical treatment procedures, including high-speed grinding, high-pressure homogenization, and ultrasonication, to produce MFC and NFC [2]. Chemical treatments, such as acid hydrolysis [3], tetracycline hydrochloride [8], and TEMPO-mediated oxidation [9], are also conducted to obtain CNC. MFC has recently gained popularity among researchers and industries owing to its attractive properties, such as high water-binding capacity, and specific surface area (m^2^/g).

Along with these properties, MFC improves on the tensile strength, thermal stability [10], and water vapor permeability of MFC-reinforced polylactic acid (PLA) for packaging materials. Nonetheless, the utilization of MFC as a food and cosmetic ingredient requires strict adherence to food safety-related guidelines because of its limitation as a hazardous chemical for humans. Therefore, mild processes such as dilute-acid pretreatment coupled with mechanical grinding are viable options in the manufacturing of food-grade MFC. In case of high-solid MFC (HMFC), such processes are required due to efficiency and transportation costs. However, reports on improvements to MFC properties, particularly at high-solid concentration, are limited [2, 11], because the fibrils agglomerate and easily lose their properties. Centrifugation is a separation process that allows the denser fraction to be sedimented and separated from the lighter liquid. It can be applied for nanoparticle separation, such as in microalgae harvesting [12], or nanomaterial purification [13]. Centrifugation is also commonly used for the concentration or dewatering process of finely dispersed particles in a liquid suspension phase [14]. Thereby, centrifugation is applied to concentrate the MFC suspension and obtain HMFC.

The term “cosmeceutical” is a portmanteau of “cosmetics” and “pharmaceuticals” and refers to cosmetic products that contain active ingredients [15]. The skin is the outermost layer of the human body and forms a barrier that protects it from innumerable environmental stresses. Among these stresses, excessive exposure to UV radiation can result in cutaneous damage, such as barrier disruption [16]. A recent trend study reported that the maintenance of skin barrier function is closely connected with skin immunity [16]. Although the skin is composed of diverse cell types, we focused on keratinocytes and macrophages, which participate in immune responses, and are well known to play fundamental roles in skin inflammation [17]. These reasons emphasize the need for a protective strategy to maintain the skin barrier function and restore homeostasis.

In this study, we prepared HMFC by the application of centrifugation and confirmed its advanced characteristics, which include higher dispersion properties and shorter fibril size. Additionally, HMFC exerts scavenging and anti-inflammatory activities via regulating HO-1/Nrf2 and MAPK pathways, respectively. Additionally, we evaluated whether high-solid MFC could be a cosmeceutical owing to its anti-oxidative and anti-inflammatory properties.

## Materials and Methods

Red algae (*Gelidium amansii*, GA) purchased from Milyang Agar (Korea), dissolved in distilled water purified by a water purification system (Direct-Q3, Merck, Germany) and sulfuric acid (Duksan; Korea), was used in this experiment. DPPH (2,2-diphenyl-1-picrylhydrazyl) was purchased from Sigma Aldrich (USA). ABTS (2,2-azinobis-[3-ethylbenzo-thiazoline-6-sulphonic acid]) was obtained from Roche (Switzerland). Inducible nitric oxide synthase (iNOS), cyclooxygenase-2 (COX-2), extracellular signal-regulated protein kinase (ERK1/2; p44/ 42), p-ERK1/2 (p-p44/42), p-JNK1/2, JNK1/2, p-p38, p38, heme oxygenase (HO)-1, and Kelch-like ECH-associated protein 1 (keap1) were purchased from Cell Signaling Technology (USA). Nuclear factor erythroid 2-related factor 2 (NRF2) and β-actin were purchased from Novus Biological (USA) and Santa Cruz (USA), respectively.

### HMFC Preparation

GA was washed, dried at 30°C for three days, milled using a blender (Hanil Electric, Korea), and filtered through a 300-μm sieve. To remove hemicellulose, 10% of GA in 1% H_2_SO_4_ was treated through a MARS 6 Microwave Digestion System (CEM Corp.; USA) with 1,800 kW power while stirring the medium at 150°C for 15 min [18]. After pretreatment, the pretreated solid was collected by filtration using a 22–25-μm filter cloth (Calbiochem; USA), and then washed with distilled water. The filter residue of the pretreated solid (PGA) was dried and used as a sample for further study.

PGA (10 g, dry weight basis) was mixed with distilled water made up to a final volume of 1,000 ml and fibrillated using a vacuum blender (Philips HR3752; China) at 37,000 rpm for 160 min, with 5 min of blending time and 25 min of cooling time to prevent the sample from overheating due to friction among the blades. MFC was centrifuged at 11,954 ×*g* and 4°C for 20 min [19] to obtain HMFC.

### Compositional Analysis

The glucan and galactan content of the biomass, pretreated biomass, and fractionated cellulose were determined following the Laboratory Analytical Procedure (LAP) of the National Renewable Energy Laboratory (NREL/TP-5100-60956) guideline, as reported earlier [2]. Monomeric sugars and organic acids in the samples were analyzed using high-performance liquid chromatography (HPLC) (Series 6000, Futecs; Korea), equipped with an Aminex HPX-87H column (300 × 7.8 mm; Bio-Rad; USA) and refractive index detector (RID), with a mobile phase of 0.01 M H_2_SO_4_, at a temperature of 50°C and a flow rate of 0.5 ml/min.

### Quantification of Dispersion Properties

The dispersion properties were determined through sedimentation rate and gel concentration point analysis. Each sample was diluted into different solid concentrations of 0.4, 0.2, 0.1, and 0.05% (w/v). The dilute sample was then vortexed and allowed to sediment for 24 h. The height of the solid was recorded at 10, 20, 30, 40, 50, and 60 min to determine the sedimentation rate. The gel concentration point (GCP) of the samples was determined at different solid concentrations by measuring the slope of the trendline from the obtained height ratio after 24 h of sedimentation [20].

### SEM and FTIR Analysis

The morphology of MFC was analyzed using a scanning electron microscope (SEM) (Hitachi SU8030; Hitachi, Japan) at a voltage of 5.0 kV. Samples were prepared with carbon tape, sputter-coated with platinum, and then visualized at a magnification of 20k×. The MFC structure was analyzed using Fourier-transform infrared spectroscopy (Nicolet iS5 FTIR Spectrometer; Thermo Scientific, USA) in the range of 4,000–500 cm^−1^ and at a resolution of 4 cm^−1^ with 32 scans in transmission mode.

### ABTS and DPPH Radical Scavenging Capacity

The radical scavenging activity of MFC was estimated using the DPPH assay. DPPH was diluted with MeOH to a concentration of 40 μg/ml, and MFC was diluted with MeOH at varying concentrations (10, 20, 40, 100, and 200 μg/ml), vortexed for 5 min, and centrifuged at 850 ×*g* for 5 min; the supernatant was then collected. In a 96-well plate, 100 μl of the MFC supernatant and diluted DPPH were added to each well. The plate was incubated at room temperature (RT, 20–25°C) for 30 min in a dark room, and then absorbance was measured at 595 nm using a microplate reader (iMark; Bio-Rad Inc.).

The antioxidant activity of MFC was also evaluated using the ABTS assay. Briefly, 7 mM ABTS and 2.45 mM potassium persulfate (K_2_S_2_O_8_) were reacted in a dark room for 12 h at RT. Subsequently, the ABTS solution was diluted in phosphate-buffered saline (PBS; Cytiva, UK) to an absorbance of 0.7 at 750 nm, and MFC was diluted with PBS as described earlier. Finally, 100 μl of ABTS solution and the supernatant were added to each well in a 96-well plate and incubated at RT for 30 min in a dark room. Absorbance was measured at 750 nm using a microplate reader (Bio-Rad). Ascorbic acid (Sigma-Aldrich) was used as a positive control.

### Cell Culture

RAW264.7 murine macrophage cells were obtained from the Korean Cell Line Bank (Korea) and maintained with a Dulbeccós modified Eagle medium (DMEM, HyClone; Cytiva) supplemented with 10% FBS (Cytiva) and 100 U/ml penicillin and 100 μg/ml streptomycin at 37°C in a humidified incubator (Thermo Fisher Scientific) with 5% CO_2_. Human epidermal keratinocyte HaCaT cells were also cultured under the same conditions as RAW264.7 cells. The cells were subcultured at 70–80% confluency and passaged with a fresh growth medium every two days.

### Cell Viability

Cell viability was quantified using 3-(4,5-dimethylthiazol-2-yl)-2,5-diphenyltetrazolium bromide (MTT)(Sigma-Aldrich) solution, which was dissolved with PBS (5 mg/ml) and stored at −20°C until further use. Subsequently, 100 μl of RAW264.7 and HaCaT cells were seeded to each well in 96-well plates at a density of 2 × 10^5^ cells/ml and 1 × 10^5^ cells/ml, respectively. The plates were incubated overnight at 37°C and 5% CO_2_. Each cell type was treated with HMFC for 24 h. After 4 h of incubation with 10 μl of MTT solution, 80 μl of the cell culture supernatant was discarded. The remaining supernatant, containing formazan, was dissolved in 100 μl dimethyl sulfoxide (DMSO, Sigma-Aldrich) for 30 min. The absorbance of the plates was measured at 595 nm using a microplate reader (Bio-Rad).

### Nitrite Assay

RAW264.7 cells were diluted at the appropriate density of 1 × 10^5^ cells/ml, and 200 μl was seeded into each well of a 96-well plate. The cells were cultured overnight in a humidified incubator and pre-treated with 12.5–100 μg/ml sample in DMEM for 1 h before exposure to 1 μg/ml lipopolysaccharide (LPS, Sigma-Aldrich) for 24 h. Subsequently, 100 μl of cell culture supernatant was incubated with Griess reagent containing 0.2% N-(1-naphthyl) ethylenediamine and 1% sulfanilamide (Sigma-Aldrich) in 5% phosphoric acid (Sigma-Aldrich) for 15 min. The absorbance of nitrite (Sigma-Aldrich) was assessed at 550 nm using a microplate reader. The calibration curve for nitrite (NO_2_^−^) in the cell culture supernatant was plotted using sodium nitrite (NaNO_2_) for the standard curve.

### ROS Production

ROS production was assessed using 2′,7′-dichlorofluorescein diacetate (DCF-DA, Sigma-Aldrich). Briefly, RAW264.7 cells (1 × 10^5^ cells/ml) were seeded on a 96-well plate and incubated at 37°C in a humidified incubator with 5% CO_2_ overnight before sample treatment. RAW264.7 cells were treated with different concentrations of the HMFC (5 and 10 μg/ml) for 1 h and then with LPS for 24 h. The culture medium in each well was discarded, cells were washed with PBS, and incubated with DCF-DA for 30 min. The cells were then washed twice with warm PBS. The fluorescence was measured using a fluorescence plate reader (SpectraMax; Molecular Devices Corporation, USA) at excitation and emission wavelengths of 485 and 538 nm, respectively. N-acetyl-L-cysteine (NAC, Sigma-Aldrich), a well-known ROS scavenger, was used as a positive control.

### Western Blot Assay

RAW264.7 cells were treated with different concentrations of the HMFC for 1 h and then with LPS or UVB. Cells were harvested after washing twice with PBS and lysed for 30 min using ice-cold cell lysis buffer (Cell Signaling Technology) containing protease and phosphatase inhibitors (Thermo Fisher Scientific). Lysates were centrifuged for 15 min at 13,000 ×*g* under ice-cold conditions. Subsequently, the supernatant was transferred into a new microtube. The protein concentration in cell lysates was calculated using a DC protein assay kit (Bio-Rad), according to the manufacturer’s instructions. Subsequently, 20–30 μg of proteins were separated on a sodium dodecyl sulfate-polyacrylamide gel (SDS-PAGE). The separated proteins in the gel were transferred to polyvinylidene difluoride (PVDF) membranes (Millipore, USA), which were then blocked with 5% skim milk for 1 h and washed with Tris-buffered saline with 0.5% Tween 20 (TBST). The monoclonal or polyclonal antibodies were used at 1:1000 dilution, and the membranes were incubated overnight with conjugated horseradish peroxidase (HRP)-linked secondary antibodies and then ECL solution (Atto, Japan). The bands were then visualized using GeneGnome XRQ NPC (Syngene; UK).

### Cytoplasmic and Nuclear Fractions

Cells were fractionated into cytoplasmic and nuclear compartments using a kit (Thermo Fisher Scientific). Briefly, RAW264.7 cells (2 × 10^5^ cells/ml) were seeded on 60- mm2 dishes and incubated overnight before HMFC treatment. Cells were incubated with an HMFC-containing medium for 1 h, then treated with 1 μg/ml LPS. After 30 min of LPS stimulation, cells were washed with ice-cold PBS. Cytosolic and nuclear proteins were extracted using Thermo Scientific NE-PER nuclear and cytoplasmic extraction kits according to the manufacturer’s instructions.

### Statistical Analysis

The experiments were performed at least thrice unless stated otherwise. For analysis, representative images from three independent experiments are depicted. Values are represented as the mean+SD of three independent experiments. *p-values of <0.05 (one-way ANOVA; Dunnett’s post-hoc test, Duncan's multiple range test, or Student’s *t*-test) were considered significant.

## Results and Discussion 

### Microfibrillation of Red Algae Biomass

The composition of the *G. amansii* polysaccharides, glucan and galactan, was approximately 24.82% and 46.53%, respectively (Table S1). Galactan was completely removed through the dilute acid pretreatment at 150°C for 15 min. This removal yield is higher compared to the cellulose increase observed in hydrothermal pretreatment at higher temperatures and longer time. This galactan removal increased the cellulose to 63.99%.

**Fig. 1:**
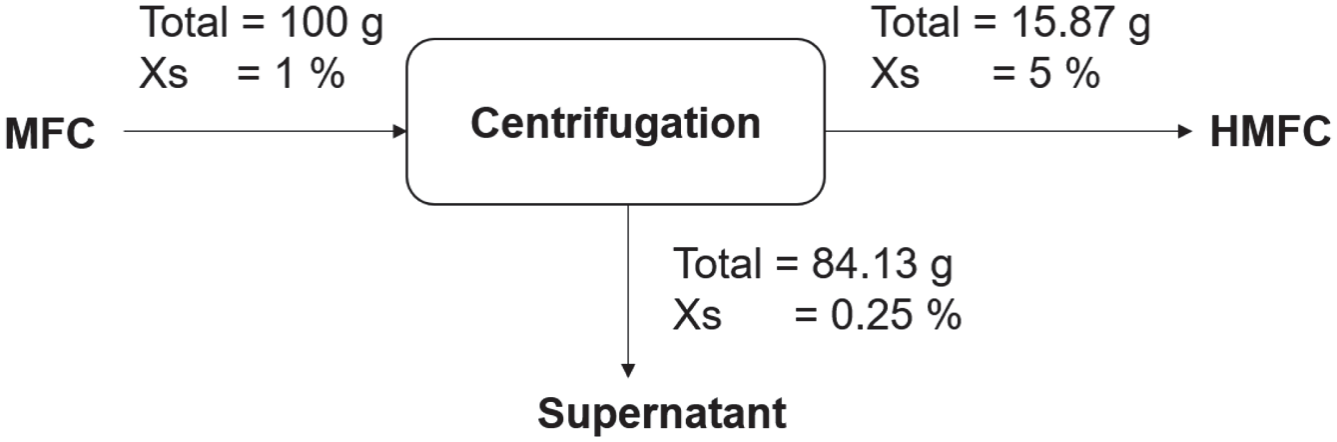
Mass balance of centrifugation.

Since the preparation of MFC is mostly conducted at lower solid concentrations [4], centrifugation was applied to concentrate the MFC into HMFC. Rotational forces from the high centrifugation speed allowed the MFC to collect at the bottom of the tube. After centrifugation for 20 min, the samples exhibited a five-fold solid concentration increase (from 1% to 5%) with solid recovery yield at 79.35% and water removal of 84.13% (Fig. 1). This result suggests that centrifugation is a suitable way of providing HMFC.

### MFC Dispersion Changes

**Fig. 2:**
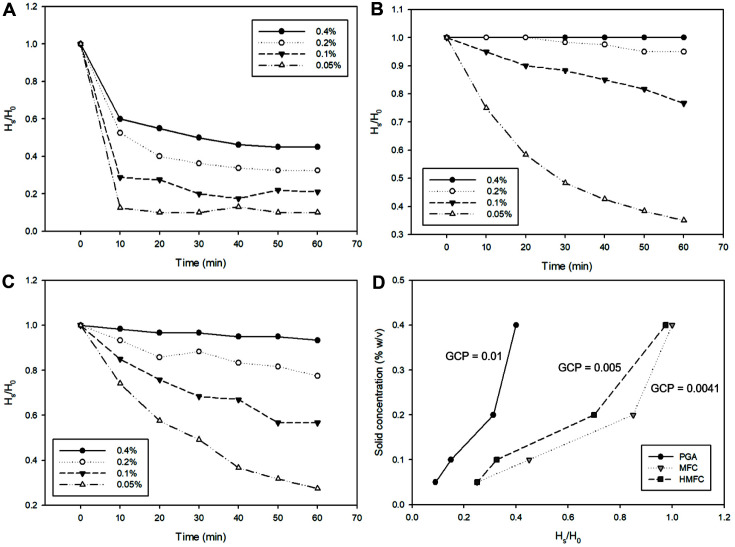
Initial sedimentation rate of extracted cellulose or PGA (A), fibrillated cellulose or MFC (B), concentrated MFC or HMFC (C), and GCP changes of cellulose along the process (D).

The dispersion properties of MFC are vital for industrial applications, such as Pickering emulsion applications in cosmetics, because these properties indirectly demonstrate the network-forming ability of MFC in the liquid system [20]. Figs. 2A–2C revealed that cellulose fibrils were entangled at solid concentrations of 0.4 and 0.2 % (w/v). The surface tension supported the cellulose in the suspension system such that it reduced the sedimentation process [21]. Counterintuitively, the sedimentation rate of MFC became faster when using lower concentrations of solids (0.1 and 0.005%, w/v) because the amount of cellulose fibrils is insufficient to allow the fibrils to entangle. Consequently, the inadequate binding of water to cellulose resulted in the gravitational sedimentation of cellulose fibrils, which can be observed in typical MFC sedimentation results.

Figs. 2A–2C also suggest that the sedimentation rate of MFC and HMFC are slower than that of PGA. It shows that blending has a significant effect on the increase in cellulose dispersion properties. Accordingly, the gel concentration point (GCP) values of MFC and HMFC sharply decreased from 0.01 to approximately 0.004 compared to that of PGA (Fig. 2D). However, the sedimentation rates of HMFC at solid concentrations of 0.02 and 0.01% are faster than that of MFC. Likewise, centrifugation slightly increased the GCP from 0.0041 to 0.005. This might be attributed to the high forces during centrifugation affecting the cellulose surface. Since the alterations in the dispersion properties of cellulose during the processing steps were observed after centrifugation, other treatments for obtaining HMFC should be further investigated.

### Morphology Changes

**Fig. 3:**
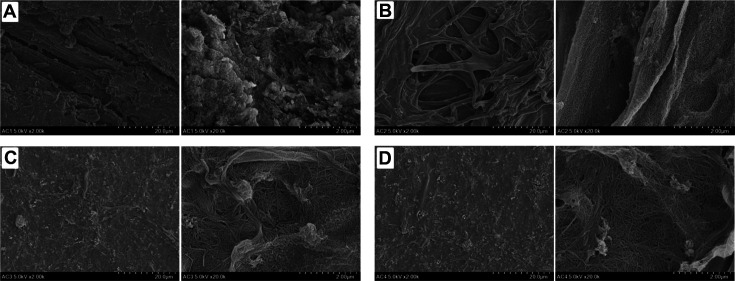
Morphology of GA (A), PGA (B), MFC 1% (C), and HMFC 5% (D); Magnification: 20 μm (left) and 2 μm (right).

**Fig. 4:**
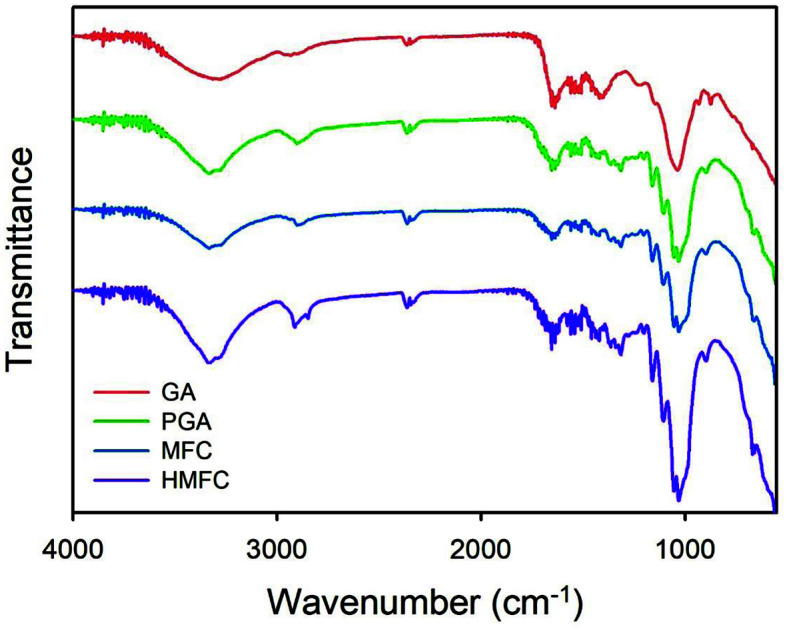
FTIR spectra of GA, PGA, MFC, and HMFC at the wavenumber range of 500–4000 cm^-1^.

Fig. 3 depicts the distinct morphology of all samples in this study. GA has a compact surface that reflects the interaction among its constituents, cellulose, hemicellulose, and other compounds. After the dilute acid pretreatment, it changes to a fibrous surface (Fig. 3B, left), demonstrating the cellulose morphology without hemicellulose interconnection. The surface of PGA also exhibits enhanced porosity and cellulose accessibility (Fig. 3B, right), probably because of alterations in biomass compositions as mentioned in general biomass pretreatments [22]. This facilitated the fibrillation of PGA to get the fibrillar structures of MFC, as shown in Fig. 3C. MFC and HMFC have significantly shorter and thinner fibrils than PGA, indicating the size reduction of fibers by microfibrillation during high-speed blending [23]. This fibril size correlates to the dispersion properties. The comparable GCP values between MFC and HMFC are also attributed to the similarity in the surface (Figs. 4C and 4D, left). However, MFC has more spaces between cellulose fibrils than HMFC to bind the water molecule, such that the GCP value of MFC is slightly lower than that of HMFC.

### Structural Changes

Fig. 4 depicts changes in the transmittance of raw GA at specific points, indicating the hemicellulose removal and cellulose intensity increase in pretreated GA and MFC, which is in accordance with the SEM image [24]. At wave number 872, 1400, and 1640 cm^-1^, transmittances were reduced because of the hemicellulose removal after acid pretreatment, although these peaks remain constant in the PGA milling and MFC centrifugation, which generally alters not chemical structures but surface morphologies. Counterintuitively, CO stretching vibration at 1030 cm^-1^ increased by higher cellulose concentration [25]. Therefore, HMFC has the highest peak at this wavenumber because centrifugation increases the solid concentration of MFC.

The CH and OH stretching vibration significantly increased at 2847 to 2900 and 3300 cm^-1^ respectively after centrifugation, suggesting that centrifugal forces were applied on the cellulose surface and the friction among cellulose fibrils allowed them to self-arrange to form a compact structure. This self-arrangement by centrifugation is also reported in the modified nanotube [26]. These structural changes proved the morphology and dispersion changes of MFC during centrifugation.

### Free-Radical Scavenging Capacity of HMFC

**Fig. 5:**
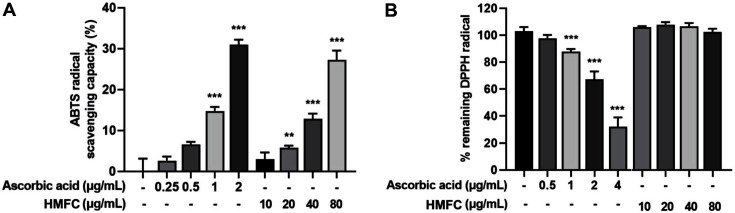
Radical scavenging activity of HMFC. The effect of HMFC on (**A**) ABTS and (**B**) DPPH radical scavenging capacity.

Owing to the harmful effect of free radicals exerted in oxidative stress conditions in biological systems, the radical scavenging activities of components play a pivotal role in radical-mediated inflammation. The effect of HMFC on radical scavenging activities was evaluated by 2,2′-azino-bis (3-ethylbenzothiazoline-6-sulphonic acid) (ABTS) and 2,2-diphenyl-1-picrylhydrazyl (DPPH) assays. Ascorbic acid has been used as a reference substance owing to its innate radical scavenger activity. The results indicated that HMFC significantly decreased the generation of ABTS free radicals (Fig. 5A); however, it did not exhibit DPPH scavenging capacity (Fig. 5B). Floegel *et al*. suggested that the ABTS assay is superior to the DPPH assay when applied to various plant-based materials containing hydrophilic, lipophilic, and high-pigmented antioxidant compounds [27]. Therefore, ABTS radical scavenging effect could be suitable for assessing MFC rather than DPPH activity.

### HMFC Suppressed LPS-Induced Inflammation and ROS in RAW264.7 Cells

**Fig. 6:**
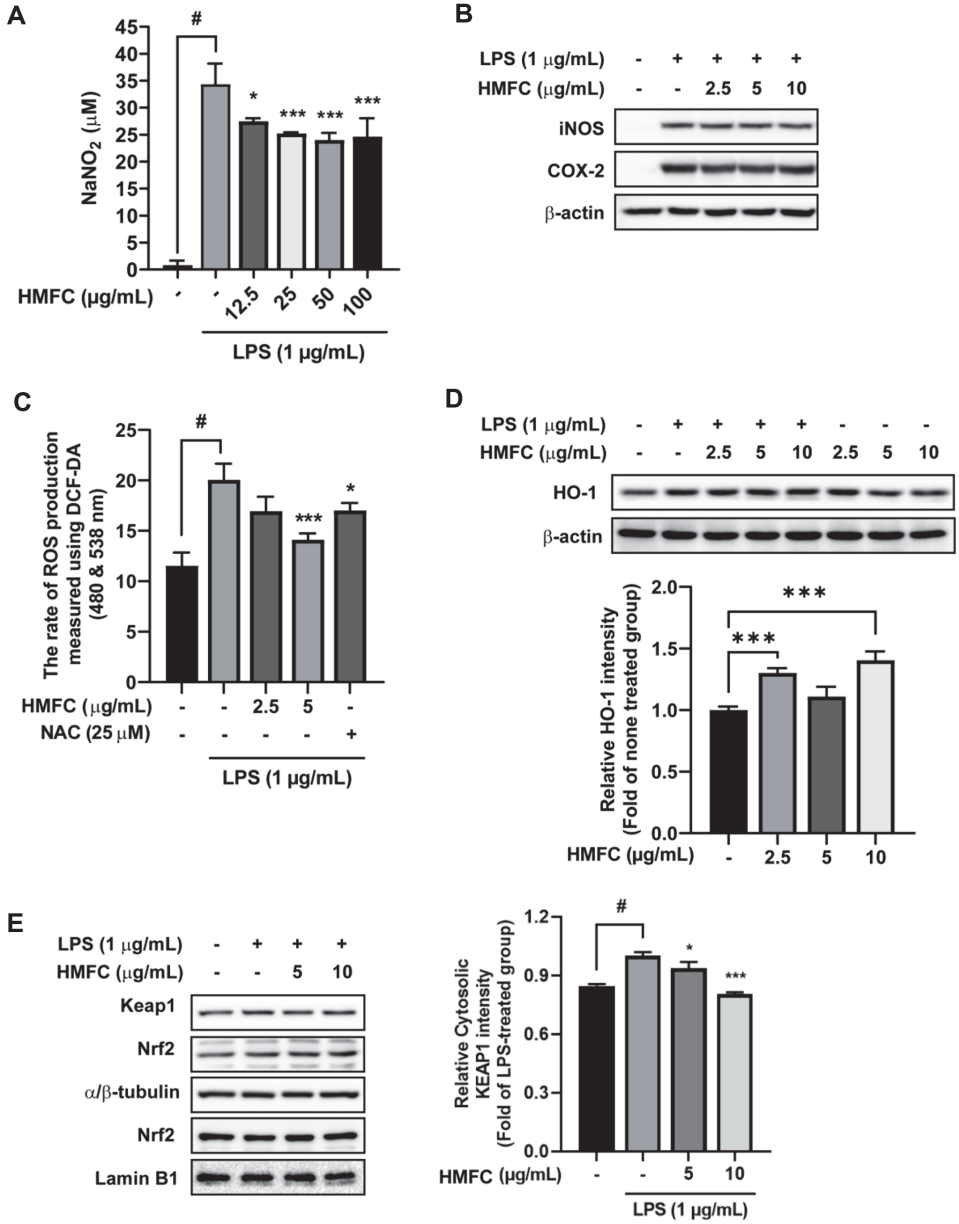
HMFC inhibits abnormal inflammation and modulates cellular oxidative stress. (**A**) HMFC attenuated LPS-induced nitrite production compared with that in the LPS-treated group. The levels of nitrite production were determined. (**B**) HMFC suppressed LPS-induced iNOS expression in RAW264.7 cells. Western blotting was used to detect alterations in iNOS and cyclooxygenase-2 (COX-2) levels. (**C**) HMFC suppressed LPS-induced excessive ROS generation in RAW264.7 cells. (**D**) HMFC upregulated HO-1 expression in RAW264.7 cells. (**E**) HMFC inhibited LPS-induced cytosolic expression of keap-1. Expression of keap-1 and Nrf2 translocation were determined using western blotting. α/β-tubulin and lamin-B1 were used as cytosolic and nuclear loading controls, respectively. The data are presented as the mean ± SD of three independent experiments. #*p* < 0.05 between the control and LPS-exposed groups; **p < 0.01 and ***p < 0.001 between LPS and HMFC- and LPS-exposed groups.

As abnormal nitric oxide production is one of the leading causes of inflammation, its modulation using marine cosmeceuticals could be a promising strategy to prevent NO-mediated inflammation. As shown in Fig. 6A, HMFC significantly suppressed LPS-induced nitrite production in a dose-dependent manner. The enzyme inducible nitric oxide synthase (iNOS) is crucial for acute inflammation because it converts L-arginine to nitric oxide [28]. Our western blot assay results revealed that HMFC suppressed LPS-induced iNOS expression in RAW264.7 cells, but not that of COX-2 (Fig. 6B).

Under severe oxidative stress, including LPS exposure, damaged cells create excessive reactive oxygen species (ROS). In addition, 2′,7′-dichlorodihydrofluorescein diacetate (DCFH-DA) was used to directly measure intracellular ROS, and N-acetyl-L-cysteine (NAC) was used for positive control because of its antioxidant activity. HMFC significantly ameliorated LPS-induced ROS production in RAW264.7 cells (Fig. 6C). Heme oxygenase (HO)-1 expression via genetic engineering and activation of nuclear factor erythroid 2-related factor 2 (Nrf2) appears to have antioxidant protection effects, and reportedly diminishes proinflammatory responses. Therefore, we investigated the antioxidant effects of HMFC and the results revealed that HMFC induced HO-1 expression (Fig. 6D). The transcription factor Nrf2 is regulated by Kelch-like ECH-associated protein 1 (keap-1), which mediates its proteasomal degradation. HMFC significantly decreased keap-1 expression but did not affect Nrf2 expression and translocation from the cytosol to the nucleus (Fig. 6E). To evaluate the cell toxicity of HMFC in RAW264.7 cells, cell viability was measured by the MTT assay. No significant difference was observed between the sample and control groups (data not shown). Collectively, we confirmed that the antioxidant effect of HMFC can reduce LPS-induced inflammation in RAW264.7 cells.

### HMFC Suppressed UVB-Induced Inflammation by Inhibition of AP-1/MAPKs Signaling Pathways in HaCaT Cells

UV radiation exposure results in DNA damage, inflammation, photoaging, and cancer in the skin [29]. UVB can function as a stress initiator followed by upregulation of COX-2, which causes prostaglandin E2 generation and subsequent inflammation [30]. We investigated whether HMFC affects UVB-induced COX-2 expression in human keratinocyte HaCaT cells. Epigallocatechin gallate (EGCG) is a well-known polyphenol derived from green tea, which inhibits UVB-induced COX-2 expression [31]. EGCG was used for positive control to confirm the repressive effect on UVB-induced COX-2 expression. However, as shown by western blotting (Fig. 6A), HMFC did not affect UVB-induced COX-2 expression. Thus, we investigated the effect of HMFC on UVB-induced phosphorylation of c-Jun, a subunit of the transcription factor activator protein 1 (AP-1). AP-1 regulates skin inflammation in response to UVB [32]. We observed that HMFC significantly attenuated phosphorylation of c-Jun at 10 μg/ml in HaCaT cells (Fig. 6B). The MAPK pathway is pivotal for the activation of c-Jun/AP-1. Because phosphorylation of c-Jun is mediated by MAPK signaling, we next investigated whether HMFC influences the phosphorylation of MAPK. Our findings indicate that HMFC improves UVB-induced phosphorylation of p38, c-Jun N-terminal kinase (JNK) 1/2, and extracellular signal-regulated kinase (ERK) 1/2 (Fig. 6C). MTT results revealed that concentrations of 100 and 200 μg/mL were cytotoxic (Fig. 6D). Overall, we demonstrated that HMFC might be a suitable cosmeceutical material against skin inflammation.

*Gelidium amansii* is a potential biomass resource for the manufacturing of MFC. Acid pretreatment could increase the glucan content of GA, which would provide high-yield cellulose. Microfibrillation successfully led to the breakdown of cellulose fibrils into smaller sizes and improved their dispersion properties. HMFC properties remain close to MFC despite undergoing high-speed centrifugation. HMFC inhibited the production of ABTS free radicals significantly. HMFC also decreased LPS-induced expression of iNOS and keap-1 in RAW264.7 cells. HMFC attenuates UVB-induced phosphorylation of c-Jun, p38, extracellular signal-regulated kinase (ERK) 1/2, and c-Jun N-terminal kinase (JNK) 1/2. Overall, we demonstrated that HMFC could be a potential cosmeceutical material for improving skin inflammation.

## Supplemental Materials

Supplementary data for this paper are available on-line only at http://jmb.or.kr.

## Figures and Tables

**Fig. 7:**
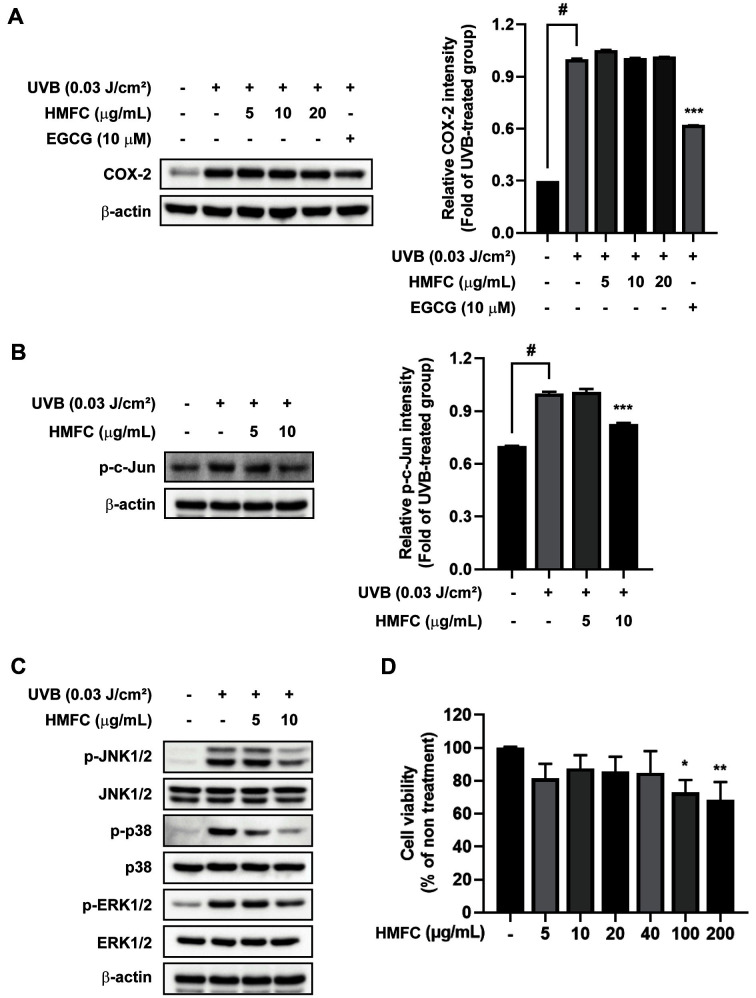
HMFC lowers the UVB-induced AP-1/MAPKs pathway in HaCaT cells. (**A**) COX-2 expression and (**B**) phosphorylation of c-Jun were detected using western blot analysis. HMFC lowered UVB-induced phosphorylation of c-Jun. The data represent the mean ± SD of three independent analyses. #*p* < 0.05 between the control and UVB-exposed groups; ***p < 0.001 between the groups exposed to UVB and HMFC and UVB alone. (**C**) The levels of phosphorylated and total ERK1/ 2, JNK1/2, and p38 after HMFC treatment and UVB irradiation were determined using western blot analysis. (**D**) Cell viability in HMFC-treated HaCaT cells was measured via MTT assay. *p < 0.05 and **p < 0.01 between the HMFC-treated and control groups.
