# Supplementary material for: Preparation of High-Solid Microfibrillated Cellulose from Gelidium amansii and Characterization of Its Physiochemical and Biological Properties
Source: J Microbiol Biotechnol. 2022 Nov 17;32(12):1589–98. doi: 10.4014/jmb.2210.10009 (PMC9843813; doi:10.4014/jmb.2210.10009)
Supplement: Supplementary file 1 [file jmb-32-12-1589-supple.pdf]

J. Microbiol. Biotechnol.
https://doi.org/10.4014/jmb.2210.10009

Supplementary Table

Table S1. Galactan removal during pretreatment

Sample	Glucan (% w/w)	Galactan (% w/w)
Raw GA	24.82±0.038[a]	46.53±0.0231
Pretreated GA	63.99±0.002[b]	N.D.

N.D., not defined; GA, *Gelidium amansii*
